# Prevalence of liver flukes infections and hydatidosis in slaughtered sheep and goats in Nishapour, Khorasan Razavi, Iran

**DOI:** 10.14202/vetworld.2018.146-150

**Published:** 2018-02-09

**Authors:** Majid Aminzare, Mohammad Hashemi, Samaneh Yaghoobi Faz, Mojtaba Raeisi, Hassan Hassanzadazar

**Affiliations:** 1 Department of Food Safety and Hygiene, School of Public Health, Zanjan University of Medical Sciences, Zanjan, Iran; 2 Department of Nutrition, Faculty of Medicine, Mashhad University of Medical Sciences, Mashhad, Iran; 3 Department of Food Hygiene and Aquaculture, Faculty of Veterinary Medicine, Ferdowsi University of Mashhad, Mashhad, Iran; 4 Cereal Health Research Center, Golestan University of Medical Sciences, Gorgan, Iran

**Keywords:** *Dicrocoeliasis*, *Fascioliasis*, *Hydatidosis*, sheep and goat, slaughterhouse

## Abstract

**Background:**

Food-borne trematode infections and hydatidosis are endemic diseases caused by helminths in Iran that are responsible for great economic loss and getting public health at risk.

**Aim:**

Aim of this study was to determine the prevalence of fasciolosis, dicrocoeliasis, and hydatidosis infections in slaughtered sheep and goats in Nishapour, Khorasan Razavi province of Iran.

**Materials and Methods:**

A survey was implemented on 130,107 sheep and goats slaughtered at an abattoir in Nishapour (Neyshbur) city, north central Khorasan Razavi Province, Iran, to determine the prevalence of fascioliasis, dicrocoeliosis and presence of hydatidosis.

**Results:**

During a 1-year period of study, among 130,107 of sheep and goats slaughtered at Nishapour abattoir, 1064 and 7124 livers were condemned totally and partially, respectively. A total of 255 (0.19%), 181 (0.12 %), and 7751 (5.95%) of livers were condemned due to cysts of *Echinococcus granulosus*, flukes of *Fasciola* spp**., and *Dicrocoelium dendriticum*, respectively. Totally, 1932 (1.48%) lungs were condemned due to hydatidosis. The significant seasonal pattern was seen for fasciolosis, dicrocoeliosis, and hydatidosis, statistically (p<0.01).

**Conclusion:**

According to this study, it seems that Neyshabour is considered as an endemic region for *Fasciola* spp. and *D. dendriticum* infections and *D. dendriticum* is the most widespread liver fluke found in sheep and goats.

## Introduction

*Fasciola* spp*., Dicrocoelium dendriticum*, and *Echinococcus granulosus* are the most common helminths that are found in many parts of the world including Iran [1,2]. Liver and lungs are important organs that are usually infected with these parasites. Parasitic infections in ruminants such as food-borne trematode infections and hydatidosis are endemic diseases caused by helminths in Iran that are responsible for great economic loss due to many disorders which results in mortality, reduction of milk production, loss of weight gain, cachexia, condemnation of livers and lungs, high susceptibility to secondary infections and getting public health at risk [3,4].

Fascioliasis (fasciolosis) and dicrocoeliasis are the two endemic parasitic diseases of Iran. Liver fluke infections (*Fasciola* spp. and *D. dendriticum*) were seen in most of herbivorous mammals such as sheep, goats, cattle, buffaloes, and human as definitive hosts for these parasites. Sheep are particularly susceptible to hepatic trematodes including *Fasciola hepatica, Fasciola gigantica*, and *D. dendriticum*. They are the most important trematodes of domestic ruminants and a common cause of liver fluke disease particularly *F. hepatica* in temperate areas of the world, Middle East including Iran [1,5-9]. Presence of freshwater snail species of the family Lymnaeidae (*Limnea* species) as intermediate host is the distribution agent of the fascioliasis disease in each area [3,10]. Several studies demonstrated the presence of fasciolosis in many provinces of Iran including Arak, Khuzestan, Mazandaran, Kurdistan, Kermanshah, Tehran, Zanjan, Azerbaijan, Gilan, and Fars [1,3,4,11,12]. Studies showed that prevalence of fasciolosis among domestic animals is higher in the southern part of Iran, but the incidence of the human disease is significantly higher in the Northern provinces [1]. Symptoms of dicrocoeliasis in domestic animals is less severe than fasciolosis, but its economic losses mainly as a result of affected liver condemnation are notable [1,13]. In a cross-sectional study during 5 years (2003-2007), Tavakoli *et al*. [14] reported that among studied provinces the highest fasciolosis and dicrocoeliasis infections rate were in Gilan (20.91%), Mazandaran (16.36%), and Esfahan (9.95%) provinces, respectively, while the lowest infection rate was seen in Ilam (0.76%), Boushehr (0.84%), and Yazd (1.51%), respectively.

*Cystic echincoccosis* (CE) or hydatid cyst known as hydatidosis is the larval form of *E. granulosus* in intermediate hosts that can also cause considerable economic losses and public health problems [15]. Studies have shown a high incidence of CE in animals in developing countries, Mediterranean, Middle East and south west of Asia including Iran in sheep, goats, cattle, buffaloes, and camels [15-19].

Due to great medical and veterinary importance of fasciolosis, dicrocoeliasis, and hydatidosis around the world including in Iran and the scarcity of information on the prevalence of these three parasitical infections in slaughtered animals in Nishapour abbatoir, Iran; therefore, the purpose of this study was to determine the prevalence of fasciolosis, dicrocoeliasis, and hydatidosis infections in slaughtered sheep and goats in Shahroud, Semnan province of Iran.

## Materials and Methods

### Ethical approval

Samples were collected from slaughtered animals.

### Study area and samples

In this cross-sectional survey, the total numbers of slaughtered sheep and goats, liver condemnations due to these three parasitical infections and both liver and lung condemnations due to hydatid cysts were recorded during March 2016–March 2017 at an abattoir in Nishapour (Neyshbur) city, north central Khorasan Razavi Province, Iran, with long periods in the range of 58° and 8 min to 59° and 20 min of longitude and 35° and 35 min to 36° north latitude and 52 min, in fact. Nishapour as the second largest city of Khorasan Razavi province situated in a fertile plain at the foot of the Binalud Mountains has a generally Mediterranean climate with the rainy seasons mostly in the spring and winter.

### Parasitological examination

Liver and lungs of 130,107 sheep and goats were inspected according to the method described by Ogambo-Ongoma [20] to recognize fasciolosis and dicrocoeliosis and for the presence of cysts of echinococcosis. The parasites were identified by their morphological characteristics [4,15]. Visualization, palpation, and incision of livers and lungs were used to extract the prevalence of these parasites. The prevalence was reported seasonally to determine the difference between distributions of infections.

### Statistical analysis

SPSS software Version 16 (SPSS Inc., Chicago, IL, USA) was used for analyzing data. One way ANOVA and chi-square tests were used to determine contamination abundance and seasonal prevalence correlation, respectively.

## Results

**Figure-1:**
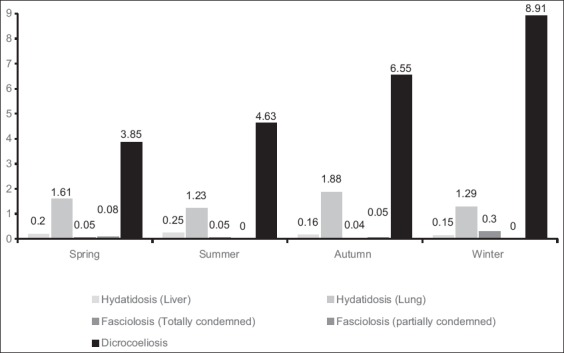
Seasonal pattern of prevalence (%) of hydatidosis (liver), hydatidosis (lung), *Fasciolosis*, and *Dicrocoeliosis* in slaughtered animals at Neyshabour abattoir during March 2016–March 2017.

During 1-year period of study, among 130,107 of sheep and goats slaughtered at Nishapour abattoir, 1064 and 7124 livers were condemned totally and partially, respectively (Table-1). A total of 255 (0.19%), 181 (0.12%), and 7751 (5.95%) of livers were condemned due to cysts of *E. granulosus*, flukes of *Fasciola* spp., and *D. dendriticum*, respectively. Totally 1932 (1.48%) of lungs were condemned due to hydatid cysts significant seasonal pattern was seen for fasciolosis, dicrocoeliosis, and hydatidosis, statistically (p<0.01) (Figure-1).

**Table-1:** Contamination abundance of fasciolosis, dicrocoeliosis, and hydatidosis in the slaughterhouse of Nishapour during March 2016–March 2017.

Season	Slaughtered	Fasciolosis (liver)		Dicrocoeliosis (liver) condemnation	Hydatidosis (liver and lung) condemnation	
		
		Totally condemned (%)	Partially condemned (%)	Totally condemned (%)	Liver (%)	Lung (%)
Spring	27188	14 (0.05)	21 (0.08)	1048 (3.85)	54 (0.2)	438 (1.61)
Summer	40030	22 (0.05)	0 (0)	1852 (4.63)	102 (0.25)	494 (1.23)
Autumn	31806	13 (0.04)	17 (0.05)	2082 (6.55)	51 (0.16)	598 (1.88)
Winter	31083	94 (0.3)	0 (0)	2769 (8.91)	48 (0.15)	402 (1.29)
Total	130107	143 (0.11)	38 (0.03)	7751 (5.96)	255 (0.2)	1932 (1.48)

## Discussion

According to obtained results, the prevalence of fasciolosis, dicrocoeliosis, and hydatidosis was high in Nishapour (Neyshbur) city. Parasites are different in the complexity of their lifecycles. Some of them complete their lifecycle in a single host (simple lifecycle parasites), while others complete it in multiple hosts (complex lifecycle parasites [CLPs]) [21]. Fasciolosis, dicrocoeliosis, and hydatidosis are CLPs, so all factors that impact on the final or intermediate host presence can increase their prevalence. Mediterranean climate with the rainy seasons mostly in the spring and winter, the presence of green pastures and the large number and variety of livestock in this region are the main reasons for high prevalence [22]. Many reports are present of the high occurrence of these zoonotic infections in many Iranian provinces, particularly in north, northwest, and northeast provinces [14,21]. The infection rate is lower in center and southern provinces of Iran due to the low population of livestock, lack of intermediate hosts of parasites and environmental harshness [14,21]. In Iran, the average prevalence of 17.8%, 19%, 11.5%, and 34.6% fasciolosis has been reported in cattle, sheep, goats, and camels, respectively [10,18]. In the present study in comparison, the lower average prevalence of (0.12%) fasciolosis in slaughtered sheep and goats and liver condemnation due to low infestation was recorded. The prevalence of liver fasciolosis was reported variously throughout the world and Iran. Infection rate of fasciolosis in Pakistan, Turkey, Nigeria, and Saudi Arabia was reported 51.3% and 14.8%, 3.99%, 9-10.3%, 0.04%, and 0.00% in sheep and goats, respectively [23-26]. Khanjari *et al*. [1], Sayadi *et al*. [11], Ansari-Lari and Moazzeni [3], and Ezatpour *et al*. [27] reported prevalence of fasciolosis in sheep 6.6%, 1.12%, 2.9%, and 6.3% in Mazandaran (Amol), Arak, Shiraz and Lorestan provinces, respectively. Goats infestation was lower than in sheep due to grazing on leaves and branches on trees and bushes whereas sheep graze on plants on the ground [3,11,28]. Availability of suitable habitat for snails as intermediate hosts, temperature and humidity are the main factors to consider in the epidemiology of fasciolosis [10].

*Dicrocoeliasis* was responsible for 5.95% of liver condemnations in this study. Prevalence of dicrocoeliosis was higher than fasciolosis (0.12%) in this study, in both sheep and goat that slaughtered in Neyshabour abattoir. These results are consistent with reports of Oryan *et al*. [29], Khanjari *et al*. [30], Mirzaei *et al*. [4], Movassagh and Valilou [31], Gargili *et al*. [24], and Ansari-Lari and Moazzeni [3], but were inconsistent with the data reported by Sayadi *et al*. [11], and Radfar and Sakha [32]. The high occurrence of dicrocoeliasis can be related to various factors such as soil type (calcareous or alkaline soils), local environmental, ecological factors, and low requirements of intermediate hosts of *Dicrocoelium* to moisture [3,29]. Sheep are more susceptible than goats to *D. dendriticum* [1,33].

Another prevalent disease in livestock of many Iranian provinces is hydatid cysts, in particular in regions with green pastures and a high number of livestock such as North, northeast, and west provinces [14,22,34]. The occurrence of hydatidosis in sheep and goats was found to be 1.67% during the study period which is lower than average prevalence of hydatidosis in animals in Iran (8.1%) [3,22]. The lowest prevalence of hydatidosis was reported in goats, probably due to the diet of goats [15]. In the present study, the prevalence of hydatidosis was higher in lungs (1.48%) than in livers (0.19%). Several studies were implemented to determine the prevalence of hydatidosis in livers and lungs of livestock throughout the world and Iran. Azami *et al*. [15], Kebede *et al*. [35], Mohamadzadeh *et al*. [36]**, Yaghan *et al*. [37], Abdi *et al*. [38], and Faraji *et al*. [39] were reported liver infection of sheep 16.4 %, 0.86 %, 2.25 %,10.6%, and 4%, respectively. A comparison between the results of the present study with the above studies reveals that the infection of hydatidosis in Neyshabour is different probably due to a different distribution pattern of the parasite. Variation in prevalence depends on several factors including strain differences of *E. granulosus* in different geographical locations, age of animal, different in culture, social activities, lack of standardized of animal health services, vicinity of animals with dogs and presence of wild carnivores such as foxes, wolves, jackals, and Hyenas [15,36,40,41].

Seasonal pattern of prevalence (%) and statistical analysis (Chi-square test) was shown a significant correlation between fasciolosis, dicrocoeliosis, and hydatidosis prevalence and seasons in this study (p<0.01) (Figure-1 and Table-2). In all animal species (sheep and goats, respectively) in this study, the highest infection rate due to *Fasciola* spp. and *D. dendriticum* was seen in the winter and the autumn and winter, respectively (Figure-1). The highest seasonal prevalence of hydatidosis especially lung form was seen in autumn and winter seasons (Figure-1). Fluke eggs hatching and their surviving ability, environmental condition, intermediate hosts multiplying and animals movement from lowland to mountain pastures where they become infected by the intermediate hosts (such as ants for dicrocoeliosis) and then bring the infection back to the valley during the winter are the main reasons of high prevalence in certain seasons [1,27,42]. Moreover, high stress induced by the transhumance on pasture-grazing nomadic sheep and goats during migratory period seems to predispose animals to infection [27,29,33].

**Table-2:** Fasciolosis, dicrocoeliosis, and hydatidosis prevalence (%) and seasonal correlation (Chi-square test).

Parasitic infection	Spring	Summer	Autumn	Winter	p value
Hydatidosis (liver)	0.20	0.25	0.16	0.15	0.0077
Hydatidosis (lung)	1.61	1.23	1.88	1.29	<0.001
Fasciolosis (totally condemned)	0.05	0.05	0.04	0.3	<0.001
Fasciolosis (partially condemned)	0.08	0	0.05	0	<0.001
Dicrocoeliosis	3.85	4.63	6.55	8.91	<0.001

## Conclusion

According to this study, it can be concluded that Neyshabour is considered as an endemic region for *Fasciola* spp. and *D. dandriticum* infection. *D. dandriticum* is the most widespread liver fluke found in sheep and goats. It seems that direct inspection method still is the best approach to estimate the prevalence of liver flukes and hydatidosis in livestock. More actions are suggested to formulate appropriate control strategies to decrease diseases and economic loss due to the condemnation of infected livers in Iran. Some treatment strategies with anthelminthic drugs and education of ranchers, safe disposal of infected offal in slaughterhouses, treatment of stray dogs as *E. granulosus* spreader are recommended. Parallel with the application of control measures; further surveys are strongly recommended collect more data about the liver flukes infection prevalence and risk factors for developing a prediction model in small ruminants in the study area and Iran.

## Authors’ Contributions

MA and MH planned and designed for the study. The Data were collected in the fields by SYF and MR. MA and HH analyzed the data and achieved statistical analysis. HH drafted and revised the manuscript. Finally, all authors read and approved the final manuscript.
